# An Unbiased Analysis of Candidate Mechanisms for the Regulation of *Drosophila* Wing Disc Growth

**DOI:** 10.1038/srep39228

**Published:** 2016-12-20

**Authors:** Jannik Vollmer, Dagmar Iber

**Affiliations:** 1 Department of Biosystems Science and Engineering (D-BSSE), ETH Zurich, Mattenstrasse 26, 4058, Basel, Switzerland; 2 Swiss Institute of Bioinformatics (SIB), Mattenstrasse 26, 4058, Basel, Switzerland

## Abstract

The control of organ size presents a fundamental open problem in biology. A declining growth rate is observed in all studied higher animals, and the growth limiting mechanism may therefore be evolutionary conserved. Most studies of organ growth control have been carried out in *Drosophila* imaginal discs. We have previously shown that the area growth rate in the *Drosophila* eye primordium declines inversely proportional to the increase in its area, which is consistent with a dilution mechanism for growth control. Here, we show that a dilution mechanism cannot explain growth control in the *Drosophila* wing disc. We computationally evaluate a range of alternative candidate mechanisms and show that the experimental data can be best explained by a biphasic growth law. However, also logistic growth and an exponentially declining growth rate fit the data very well. The three growth laws correspond to fundamentally different growth mechanisms that we discuss. Since, as we show, a fit to the available experimental growth kinetics is insufficient to define the underlying mechanism of growth control, future experimental studies must focus on the molecular mechanisms to define the mechanism of growth control.

A fascinating aspect of embryonic development is how the growth of organ rudiments is globally coordinated such that all organs, tissues and the paired appendages grow to the correct (relative) size, even when final sizes differ between isogenic offspring because of external factors such as nutrition or temperature^1^. The mechanism by which growth is terminated has remained elusive.

Growth control has been studied extensively in *Drosophila* imaginal discs and can be separated into disc-intrinsic and disc-extrinsic mechanisms. Disc-extrinsic mechanisms ensure that body growth and organ growth are coupled in a way that correct size proportions are obtained, even when nutritional status and thus final size differs strongly during development, a phenomenon referred to as allometry^2^,^3^. Intriguingly however, similar final imaginal disc sizes are obtained even when imaginal discs are provided with additional developmental time^4^,^5^, when the frequency of cell divisions is perturbed^6^,^7^, after regeneration of lost tissue^8^,^9^, and when imaginal discs are cultured outside the larvae^10^,^11^. These results point to the existence of a disc autonomous mechanism of size control that ensures correct final sizes.

The morphogen Decapentaplegic (Dpp) affects growth in all fifteen imaginal discs in the *Drosophila* larvae, and models have been suggested how Dpp may result in a uniform declining growth rate^12^,^13^,^14^,^15^,^16^,^17^,^18^ in spite of its graded distribution in imaginal discs^19^,^20^,^21^. In the wing disc, in particular, Dpp forms a dynamically increasing gradient^17^ from the anterior-posterior boundary across the anterior and posterior sides^22^,^23^,^24^,^25^. In the eye disc, on the other hand, Dpp is secreted from the morphogenetic furrow (MF) that sweeps from the posterior to the anterior end of the eye disc^26^,^27^. Gonzalez-Gaitan and co-workers previously proposed that an exponential decline in the proliferation rate arises in both discs because cells sense the relative local change in the Dpp concentration and divide whenever they experience a fixed relative change of about 40%^17^,^28^. However, the area growth rate is unaltered in Mad-/Brk- clones that cannot sense Dpp^28^,^29^. Additionally, Dpp affects wing disc growth only in the first half of larval development^30^ and only in the medial part of the wing disc^31^. According to an alternative model, uniformly declining growth results from a combination of Dpp signaling and mechanical feedbacks^12^,^15^,^32^,^33^. Finally, we have recently shown that the growth rate of the apical area in the *Drosophila* eye imaginal disc declines inversely proportional to the total apical area, which is consistent with a dilution-based mechanism for growth control^34^.

Here, we use published quantitative growth data to carry out an unbiased evaluation of alternative candidate growth mechanisms. Based on the quantitative analysis, we rule out growth control by dilution in the wing disc. Evaluating alternative candidate growth mechanisms, we find that biphasic exponential growth best fits the wing disc data. An exponential decline of the growth rate with developmental time and logistic growth are also consistent with the data. These three growth laws correspond to fundamentally different growth mechanisms that we discuss. Given the wide range of growth laws that are consistent with the data, we emphasize the need to explore a wider range of growth limiting mechanisms and to carefully check the consistency of any proposed mechanism with additional experimental data – the ability to recapitulate the growth kinetics alone provides insufficient support for any mechanism.

## Results and Discussion

### Dilution of a cytokine cannot explain the Wing Disc Growth Kinetics

The growth of the apical area, *A*, of *Drosophila* imaginal discs over developmental time, *t*, can be described mathematically by


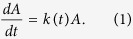


Here, *k* refers to the area growth rate. We have previously shown that the declining area growth rate in the eye disc can be described by an area-dependent decline^34^ of the form


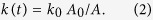


**Figure 1:**
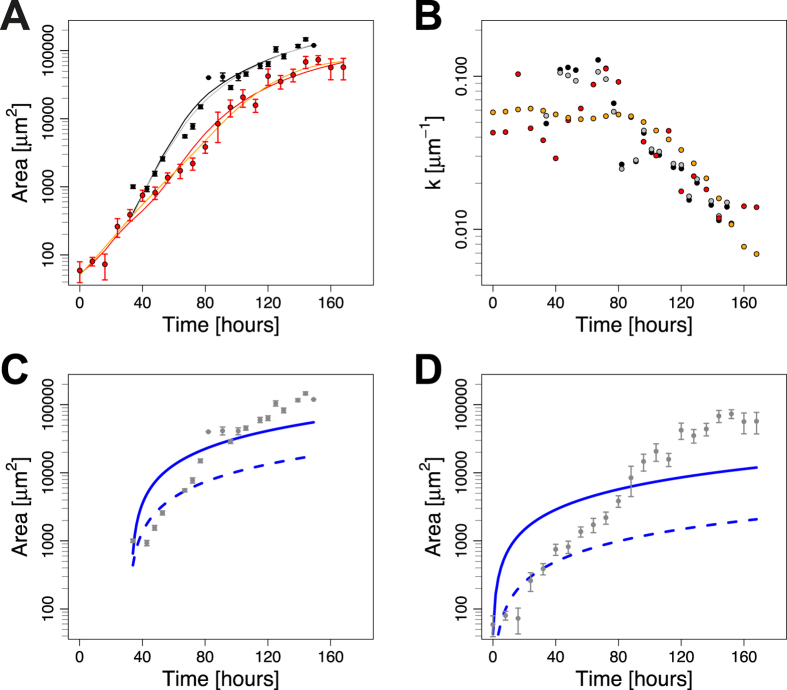
A dilution mechanism cannot explain growth control in the *Drosophila* wing disc. (**A**) *Drosophila* wing disc area growth as reported in ref. 17 (black) and ref. 35 (red). Two spline fits (lines, see Materials and Methods for details) were fitted to each data set to estimate the slope at each data point. (**B**) The area growth rate *k* as determined from Eq. (8) declines over developmental time. Colour code corresponds to the data and fits in panel A. (**C**,**D**) A model based on the dilution mechanism (Equation 2) fits the two independent wing disc growth datasets by Wartlick *et al*.^17^ (**C**), and by Nienhaus *et al*.^35^ (**D**) badly; fits were obtained using the residuals given by Eq. (9) (solid lines) or Eq. (10) (dashed lines). Parameters are given in Table S1.

Here *A*_*0*_ is the initial area and *k*_*0*_ the maximal growth rate. We sought to test whether this mechanism would also apply to the wing disc. To this end, we used two independent, published datasets for wing disc area growth^17^,^35^ (Fig. 1A) to determine the area growth rate, *k* (Fig. 1B), and to fit the model (Equations 1 and 2) to these datasets (Fig. 1C,D). The poor fit to the data rejects this mechanism for growth control in the wing disc (Fig. 1C,D, Figs S1A–D and S2A–D).

### Unbiased Analysis of Candidate Growth Laws for Wing Disc Growth

**Figure 2:**
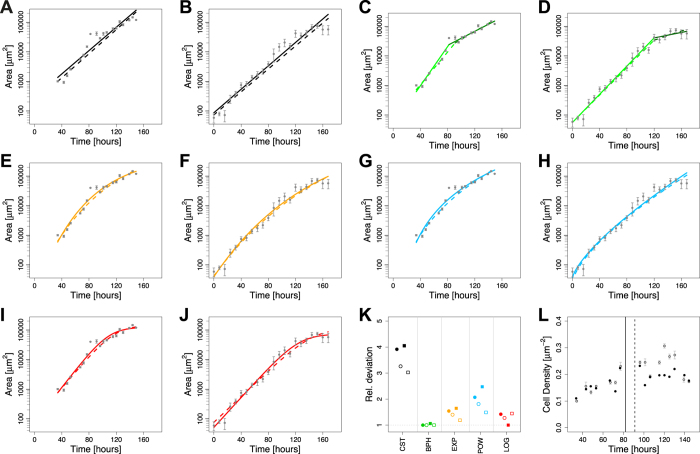
Evaluation of Candidate Growth Laws for the *Drosophila* Wing Disc. (**A–J**) Fits of the growth model in Eq. (1) with the different growth laws given by Eqs (3, 4, 5, 6) reveals best fit to the datasets obtained by Wartlick *et al*.^17^ (columns 1 and 3) and Nienhaus *et al*.^35^ (columns 2 and 4) with biphasic growth (C,D - green), logistic growth (I,J - red) and an exponentially declining growth law (E,F - yellow). The worst fit, but still better than the one based on the dilution mechanism (Fig. 1), is obtained with a constant growth law (A,B - black). A powerlaw decline (G,H - blue) provides an intermediate fit to the data. Fits were obtained using the residuals given by Eq. (9) (solid lines) or Eq. (10) (dashed lines). (**K**) Relative deviation of the resulting fits from the data (circles - Wartlick *et al*.^17^; squares - Nienhaus *et al*.^35^). The deviation was normalized with respect to the minimal value for each dataset and the residual definition (Equation 9 (closed symbols) & 10 (open symbols)). (**L**) Cell density in the wing disc over time as measured in Wartlick *et al*.^17^ (grey) and as inferred from the cell number and area data in Wartlick *et al*.^17^ (black). Vertical lines indicate the switch points that minimize the deviation between data and BPH model for the two different definitions of the residuals (Fig. S3A,B; solid line - Equation 9; dashed line - Eq. 10).

We next tested which other candidate growth laws would allow us to reproduce the wing disc data (Fig. 2). To this end, we simulated the growth model in Eq. (1) with the growth laws that we had previously considered as candidates for growth control in the *Drosophila* eye disc^34^. In addition, we considered biphasic growth, which had previously been shown to describe wing disc growth^2^, and logistic growth, which had previously been shown to describe the regeneration kinetics of newt limbs^14^. In summary, in the simplest model we assumed a constant growth rate (CST, Fig. 2A,B)


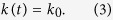


In biphasic growth (BPH), the value of *k*_0_ changes once, thus giving rise to two phases of different exponential growth (biphasic, BPH, Fig. 2C,D). The switch point from phase I to II was defined as the one providing the lowest deviation from the data (Fig. S3). In addition, we considered models with a continuously declining growth rate, either as exponential decaying growth rate (EXP, Fig. 2E,F) of the form


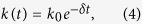


or by following a power law decay (POW, Fig. 2G,H) of the form


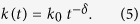


Finally, the growth rate could depend on the total wing disc area. Besides dilution (Equation 2, Fig. 1C,D), logistic growth (LOG, Fig. 2I,J) can result in an area-dependent growth rate


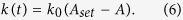


We note that the models with a constant or an area-dependent growth rate have two parameters, the biphasic exponential model (BHP) has four parameters, while all other models (EXP, POW, LOG) have three independent parameters. A full list for the parameter values can be found in the supplementary information (Tables S1 & S2).

To fit the models to the data, we defined the residuals in two independent ways: either by normalizing with respect to the standard error (dashed lines in Figs 1C,D & 2A–J & Fig. S1) or by taking the logarithm of the data (solid lines in Figs 1C,D & 2A–J & Fig. S2) to correct for the different orders of magnitudes in the data and thus be able to fit the data well (Fig. 2A–J) (see Material & Methods for details). To compare the model fits, the sum of squared residuals (RSS, Fig. 2K, Table S3) and coefficient of determination (*R*^*2*^, Table S4) were used. We note that fitting the logarithmic data provided the smaller deviation and better coefficient of determination (*R*^*2*^) in all cases (Tables S3 and S4). Using these measures, the biphasic model (BHP) performs best for almost all weightings and data sets (Fig. 2K). Interestingly, also the reported change in cell density^17^ correlates with the predicted switch point. Thus, the cell density increases until the inferred switch point (vertical lines) and subsequently stabilizes (Fig. 2L). Logarithmic growth (red) and to a slightly lesser extent an exponentially declining growth rate (yellow) also fit the data very well (Fig. 2K), and the difference to the biphasic model is only minor (Tables S3 & S4). The power law model fits the data slightly, but consistently, worse as judged by the *R*^*2*^ value (Table S4) and the RSS (Table S3).

The models with a constant (CST) or an area-dependent growth rate both have only two free parameters, and thus at least one parameter less than the models discussed above (BHP, EXP, POW, LOG). The model with the area-dependent growth rate performs considerably worse than the model with a constant (CST) growth rate, and can thus be rejected (Fig. 2K, Tables S3 & S4). To compare the model with a constant (CST) growth rate to the other models, the different number of free parameters needs to be taken into account. Unlike other commonly used model selection criterions, such as the Bayesian information criterion (BIC) or the Akaike information criterion (AIC), the F-test provides a p-value on the null hypothesis that the better fit can be solely explained by the increased number of parameters. Its use is, however, restricted to the case where the simpler model is nested within the more complex model, limiting it to the comparison of the biphasic (BHP) and single exponential model (CST). Conducting an F-test, we find that for both data sets and weightings, the biphasic exponential model fits the data significantly better than the model with a constant growth rate (Table S5).

### Candidate Mechanisms for Growth Control in the Drosophila Wing Disc

While the biphasic growth law, logistic growth, and an exponentially declining growth rate all fit the data well, they point to very different underlying mechanisms. A biphasic growth law would require a sudden change in cell growth, potentially because of cell differentiation. It has been argued that larvae can monitor their size and trigger the switch to lower growth rates when reaching a ‘critical size’^2^. We note that in contrast to models with continuously declining growth rates, the biphasic growth law would still require an additional mechanism to ultimately stop growth. Otherwise, growth would continue with the speed from the second phase. Growth in the models with a continuously declining growth rate will eventually be so close to zero that the expansion becomes negligible.

An exponential decline in the growth rate could be achieved by the Dpp-dependent mechanism proposed by Gonzalez-Gaitan and co-workers^17^,^28^, or if a growth factor to which the system responded linearly, was degraded at a constant rate δ, i.e.


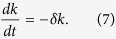


However, in both cases there are fundamental problems such as the reliable read-out at low concentrations and the robustness of the mechanism to changes in the total developmental time as observed in grafting experiments^10^,^11^. Thus, as the exponentially declining growth rate, k (Equation 4), declines 3-fold and 5-fold in the two different datasets from early to late stages^17^,^35^, cells would need to be able to linearly respond to 3–5-fold changes in this growth-controlling factor. Recent experiments indeed question a central role of the Dpp gradient in determining final disc size^29^,^30^,^31^.

Logistic growth requires a mechanism to set a final size and to reduce the growth rate as this final size is approached. The intercalation model results in logistic growth^14^. Here, the growth rate is postulated to be proportional to the positional difference between neighbouring cells, which is reduced as the structure grows out. How such positional differences would be measured by cells is not known. While loss of the cell-adhesion mediating cadherin Fat has been shown to enhance growth in the medial part of the wing disc, it has remained unclear whether the Fat-dependent growth limitation is central to growth termination^33^,^36^,^37^,^38^. Beyond its molecular implementation, an intercalation mechanism has a number of conceptual limitations. Thus, while the intercalation mechanism can explain restoration of missing positional values during regeneration and a logistic growth law would be robust to changes in developmental speed, it remains unclear how the positional identity would scale when tissues grow to different final sizes, for instance because of differences in nutrients available^2^. Finally, while it has been argued that larvae can sense a ‘critical size’^2^, it remains unclear how wing discs would achieve this on the molecular level in a disc-autonomous way and how disc size could then vary in response to changes in external conditions (nutrients, temperature etc.).

In conclusion, we can rule out an area-dependent dilution mechanism for growth control in the wing disc. Rather the responsible mechanism must give rise to biphasic growth, logistic growth, or an exponentially declining growth rate. Given that several growth laws match the measured growth curves, it needs to be stressed that the reproduction of the growth kinetics alone is insufficient evidence for any proposed mechanism and mechanistic proof needs to be provided. At the same time, there could be other growth laws that also reproduce the data and that we have not yet identified. It will be interesting whether the same mechanism limits growth in all organs and species or whether distinct mechanisms have evolved^17^,^28^,^34^,^39^,^40^.

## Materials and Methods

### Software

All simulations and analysis were done using Matlab R2016A (The MathWorks, Natick, MA, USA) or the free software environment R in version 3.2.4.^41^.

### Estimation of area growth rate

The area growth rate


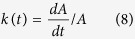


in Eq. (1) can be determined by estimating the slope 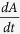 at all time points and diving this slope by the measured area *A* at each timepoint. To estimate the slope 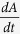 from the data without making any assumption about the (true) function underlying the growth dynamics, we used cubic smoothing splines as provided by the *smooth.spline* function in R^41^. As the exact shape of the decline of the area growth rate *k* depends on the spline fits, we used two different fits for each data set with a varying smoothing parameter. The resulting spline fits are shown in Fig. 1A, and the estimated area growth rate *k(t*) in Fig. 1B.

### Model Fitting

The two available independent studies do not provide the variance for all data points^17^,^35^. We therefore quantified the deviation of the model from the data in form of the residual *R* at time point *k* as


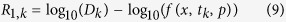


or by weighting the difference by the given standard error (SE) as


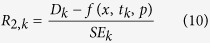


where *D*_*k*_ is the mean, *SE*_*k*_ its standard error, and *f(x,t*_*k*_*,p*) refers to the model value at time point *k*. We took the logarithm of the data for the first measure since the data is spread over several orders of magnitude and larger values would therefore be weighted much higher otherwise. In the data set from Wartlick *et al*. no SE was given for the time point at approximately 80 hours and for the last time point (SE = 0)^17^. We therefore excluded these two time points from the analysis with Eq. 10.

The model parameters were then determined by solving the minimization


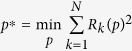


where *N* is the number of distinct time points. This was done independently for both definitions of the residuals using the trust-region reflective algorithm as implemented in *lsqnonlin* (Matlab R2016A, MathWorks, Natick, MA, USA), resulting in two parameter sets for each model.

### Comparison of model fits and statistical analysis

Models were compared based on the residual sum of squares (RSS) and their *R*^*2*^ value. The *R*^*2*^ value of model *j* was calculated as


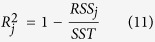


where *RSS*_*j*_ is the residual sum of squares of model *j* calculated as explained above (Equations 9 or 10) and *SST* is the total sum of squares.

To evaluate whether there is a statistical significance in the difference how the models fit the data, the F-test was used. The F-test can only be used to compare a simple model which is nested within a more complex one. Thus, here it can only be used to compare the biphasic exponential model to the single exponential model, as the latter one is nested within the first one. The F-statistic was calculated as


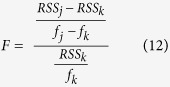


where model *j* is nested within model *k, f*_*j*_ and *f*_*k*_ are the degrees of freedom (number of data points minus number of parameters) for model *j* and *k*, respectively, and RSS their residual sum of squares. The respective p-value can then be calculated from the F cumulative distribution function. The degrees of freedom to be used in the cumulative distribution function are (*f*_*j*_ − *f*_*k*_, *f*_*k*_).

## Additional Information

**How to cite this article**: Vollmer, J. and Iber, D. An Unbiased Analysis of Candidate Mechanisms for the Regulation of *Drosophila* Wing Disc Growth. *Sci. Rep.* **6**, 39228; doi: 10.1038/srep39228 (2016).

**Publisher's note:** Springer Nature remains neutral with regard to jurisdictional claims in published maps and institutional affiliations.

## Supplementary Material

Supplementary Information
