# Supplementary material for: An Unbiased Analysis of Candidate Mechanisms for the Regulation of Drosophila Wing Disc Growth
Source: Sci Rep. 2016 Dec 20;6:39228. doi: 10.1038/srep39228 (PMC5172366; doi:10.1038/srep39228)
Supplement: Supplementary Information [file srep39228-s1.pdf]

An Unbiased Analysis of Candidate Mechanisms for the Regulation of *Drosophila* Wing Disc Growth

Supplementary Information

Jannik Vollmer[1,2], Dagmar Iber[1,2]*

Affiliations

[1]Department of Biosystems Science and Engineering (D-BSSE), ETH Zurich, Mattenstrasse 26, 4058, Basel, Switzerland

[2]Swiss Institute of Bioinformatics (SIB), Mattenstrasse 26, 4058 Basel, Switzerland

SUPPLEMENTARY TABLES

Table S1: Model parameters for all models with a constant or continuously declining growth rate, datasets and residuals definitions

Model	k(t)	Dataset	Residuals (Eq. no)	A_0 [μm^2]	k_0 [h^{-1}]	δ [h^{-1}] / x_{set} [μm^2]
P_A	$k_0\ {}^{A_0}/_{A}$	[1]	9	652.3622	0.7251	-
			10	438.7364	0.3415	-
		[2]	9	42.6635	1.6611	-
			10	31.5019	0.3922	-
CST	k_0	[1]	9	1.3603e+03	0.0453	-
			10	998.9830	0.0467	-
		[2]	9	87.2729	0.0455	-
			10	73.9243	0.0446	-
EXP	$k_0 e^{-\delta t}$	[1]	9	591.2675	0.1602	0.0145
			10	640.3194	0.1110	0.0097
		[2]	9	39.6393	0.0787	0.0069
			10	42.4253	0.0720	0.0058
LOG	$k_0(A_{set} - A(t))$	[1]	9	761.7601	5.4103e-07	1.2520e+05
			10	755.3948	4.2605e-07	1.4599e+05
		[2]	9	51.1345	7.8364e-07	7.3946e+04
			10	75.7238	4.3474e-07	1.1532e+05
POW	$k_0\ t^{-\delta}$	[1]	9	572.1909	4.5282	1.0407
			10	613.4473	1.1274	0.7155

		[2]	9	36.3991	0.1445	0.2753
			10	25.6553	0.1746	0.3211

Table S2: Model parameters for biphasic exponential model (BPH)

Dataset [REF #]	Residuals (Eq. no)	A_0 [μm^2]	k_1 [h^{-1}]	k_2 [h^{-1}]	Switch point [h]
[1]	9	615.6335	0.0762	0.0273	82.1
	10	720.5852	0.0637	0.0310	91.1
[2]	9	56.3905	0.0548	0.0107	120.0
	10	56.6731	0.0533	0.0178	120.0

Table S3: Residual sum of squares (RSS) for the different models

Dataset [REF #]	Residuals (Eq. no)	CST	BPH	POW	EXP	LOG	A-D
[1]	9	0.84	0.21	0.38	0.33	0.30	2.53
	10	271.36	83.18	137.54	116.55	106.16	1562.00
[2]	9	1.02	0.27	0.62	0.41	0.25	8.82
	10	72.95	24.11	35.90	28.6	34.84	247.83

Table S4: Coefficient of determination (R^2) for the different models

Dataset [REF #]	Residuals (Eq. no)	CST	BPH	POW	EXP	LOG	A-D
[1]	9	0.91	0.98	0.96	0.97	0.97	0.74
	10	0.89	0.96	0.94	0.95	0.96	0.34
[2]	9	0.96	0.99	0.97	0.98	0.99	0.62

	10	0.79	0.93	0.90	0.92	0.90	0.30

Table S5: P-values for F-test statistics

The F-test was used to compare the fit of the single exponential model (CST) to the biphasic exponential model (BHP). The F-statistics and p-values were calculated as explained in the Materials & Methods. P-values below 0.05 were considered significant.

**Dataset [REF #]**	**Residuals (Eq. no)**	**p-value**
[1]	9	1.22E-04
	10	4.59E-04
[2]	9	1.77E-04
	10	7.49E-04

SUPPLEMENTARY FIGURES

Figure S1. Analysis of the quality of the fits for the residuals defined as the difference between the logged data and simulation results (Eq. 9).

Column 1: Residuals for the different models plotted against the respective time-dependent data point (Data from Wartlick et al.[1]). Residuals here are defined as the difference between the logged data and simulation results. Optimization was done by minimizing the sum of the square of those values. In an optimal case, residuals should be centred around 0 (grey line). **Column 2**: Quantile-quantile-plot (qq-plot) for the residuals from column 1 compared to the ones expected for a standard normal distribution. Ideally, the values should fall onto a line. **Columns 3,4**: Residuals and qq-plot for the different models fitted to the data from Nienhaus et al.[2].

Model abbreviations are as follows: CST, constant growth rate (Eq. 3); EXP, exponentially declining growth rate (Eq. 4); POW, growth law declining according to power law (Eq. 5); LOG, logistic growth law (Eq. 6); BPH, biphasic growth law

Figure S2. Analysis of the quality of the fits for the residuals expressed as the difference between data and simulation results normalized by the standard error (Eq. 10).

Column 1: Residuals for the different models plotted against the respective time-dependent data point (Data from Wartlick et al.[1]). Residuals here are defined as the difference between data and simulation results normalized by the standard error (Eq. 10). Optimization was done by minimizing the sum of the square of those values. In an optimal case, residuals should be centred around 0 (grey line). **Column 2**: Quantile-quantile-plot (qq-plot) for the residuals from column 1 compared to the ones expected for a standard normal distribution. Ideally, the values should fall onto a line. **Columns 3,4**: Residuals and qq-plot for the different models fitted to the data from Nienhaus et al.[2].

Model abbreviations are as follows: CST, constant growth rate (Eq. 3); EXP, exponentially declining growth rate (Eq. 4); POW, growth law declining according to power law (Eq. 5); LOG, logistic growth law (Eq. 6); BPH, biphasic growth law

Figure S3: Switch-Point Analysis for the biphasic model for the different data sets and residual definitions

To define the switch point from the 1st to the 2nd phase of the biphasic growth model, all data points were screened as possible switch points and the minimal model deviation, defined as sum of the squared residuals, between the data (A,B: Wartlick et al.[1]; C,D: Nienhaus et al.[2]) and the biphasic exponential model was recorded for each of them. The switch points for each

data set (A,B: Wartlick et al.[1]; C,D: Nienhaus et al.[2]) and for each optimization routine (A,C: optimization with respect to the log-transformed data according to Eq. 9; B,D: minimization of SE-normalized residuals according to Eq. 10) presented in the main text were chosen as the ones providing the lowest deviations from the data (marked in red). The corresponding fits are shown in Fig. 2C and D.

(A) Optimization with respect to the log-transformed data according to Eq. 9. Data from Wartlick et al.[1].

(B) Optimization with respect to the minimization of SE-normalized residuals according to Eq. 10. Data from Wartlick et al.[1].

(C) Optimization with respect to the log-transformed data according to Eq. 9. Data from Nienhaus et al.[2].

(D) Optimization with respect to the minimization of SE-normalized residuals according to Eq. 10. Data from Nienhaus et al.[2].

References

1. Wartlick, O. *et al.* Dynamics of Dpp signaling and proliferation control. *Science* **331,** 1154–1159 (2011).

2. Nienhaus, U., Aegerter-Wilmsen, T. & Aegerter, C. M. In-vivo imaging of the Drosophila wing imaginal disc over time: novel insights on growth and boundary formation. *PLoS One* **7,** e47594 (2012).
